# Heat flux concentrators based on nanoscale phononic metastructures†

**DOI:** 10.1039/d3na00494e

**Published:** 2023-09-11

**Authors:** Jian Zhang, Haochun Zhang, Weifeng Li, Gang Zhang

**Affiliations:** a School of Energy Science and Engineering, Harbin Institute of Technology Harbin 150001 China hczhang@hit.edu.cn; b Institute of High Performance Computing, Agency for Science, Technology and Research (A*STAR) Singapore 138632 Singapore zhangg@ihpc.a-star.edu.sg; c School of Physics & State Key Laboratory of Crystal Materials, Shandong University Jinan 250100 Shandong China

## Abstract

In recent years, nanoscale heat flux regulation has been at the forefront of research. Nanoscale heat flux concentration is of potential importance in various applications, but no research has been conducted on local heat flux concentration. In this paper, we designed two heat flux concentrators using patterned amorphous and nanomesh structures, respectively. Using molecular dynamics simulation, we find that the heat flux in the central regions is much higher than that in the adjacent regions, with the concentration ratio arriving at 9-fold. Thus a heat flux concentrator is realized using these nanophononic metastructures. The phonon localization theory was used to explain the underlying mechanism. This work provides a direct design strategy for thermal concentrators using practical nanofabrication technologies.

## Abstract 2

In recent years, nanoscale heat flux regulation has been at the forefront of research.

## Graphical abstract

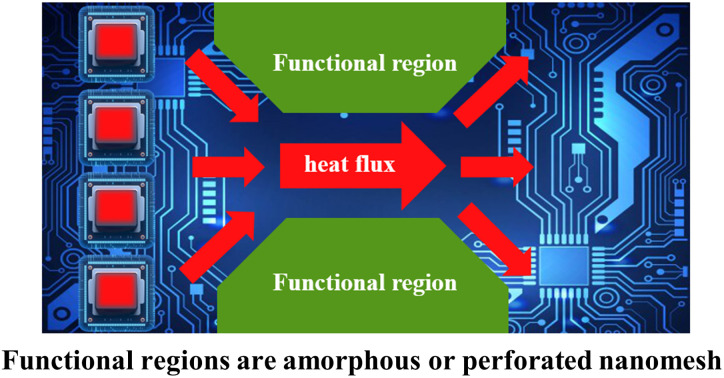

## 1 Introduction

Manipulating thermal conduction at the nanoscale has attracted great interest^1–3^ due to its critical role in understanding the fundamental physics of phonons^4^ and developing controlling strategies in various areas of application, including nanoscale thermal management,^5,6^ thermoelectrics,^7,8^ and thermal protection.^9^ A countless number of theoretical and experimental studies have been conducted to explore the various effects on the thermal conductivity of nanoscale materials, including size, strain, defects, bonding strength, and geometry configuration.^10–20^ For example, the thermal conductivity of silicon nanowires is about two orders of magnitude lower than that of bulk silicon^21^ and increases with nanowire length.^22^ And thermal conductivity of multi-layer graphene depends significantly on the thickness, and interlayer phonon scattering plays a critical role in the in-plane thermal conductivity.^23^ Overall, most of these factors affect the thermal conductivity of materials *via* the material growth process.

On the other side, after the materials are synthesized, their thermal conductivity can be further changed using the concept of phononic metamaterials (or metastructures).^24–27^ For example, the thermal conductivity of silicon nanoscale films can be reduced using periodic nanomesh of a holey structure, making it a promising candidate for thermoelectric applications.^28,29^ In periodic nanomesh, the periodicity of a hole array can induce the folding of the Brillouin zone, thus depressing phonon group velocity and reducing thermal conductivity. In experiments, the feature size of nanomesh is typical of about a few hundred nanometers, whose structure can be easily accessible with standard fabrication techniques, including electron beam lithography and reactive ion etching. Because of the development of the silicon-on-insulator technology, high-quality single-crystal silicon nanofilms with thicknesses down to a few nanometers^30^ are usually employed to fabricate two-dimensional phononic metastructures^31^ with tunable thermal conductivity.^32^ In addition to the electron beam, high-energy ion irradiation is also adopted to change the thermal conductivity of nanomaterials. This method can induce amorphization within the desired region, without causing significant voids, providing an ideal platform to explore phonon transport in nanoscale amorphous materials.^33–35^

These recent advancements in nanofabrication technologies have improved our ability to control thermal conductivity at the nanoscale, providing exciting opportunities to develop a large variety of thermal devices. The challenges in thermal management in nanoscale devices and renewable energy conversion have sparked great research interest in developing thermal/phononic devices,^36^ including thermal rectifiers,^37–39^ thermal transistors,^40^ thermal modulators,^41^ and ray phononics.^42–45^ Clearly, it is of both scientific significance and technological impact to realize novel thermal devices based on realizable nanofabrication technology.

In the present work, we designed a nanoscale heat flux concentrator by performing molecular dynamics simulations. We demonstrated the feasibility and functionality of this thermal concentrator, and we also identified the physical mechanism. Our results provide useful guidelines for the design of novel nanoscale thermal devices.

## 2 Calculation method

**Fig. 1:**
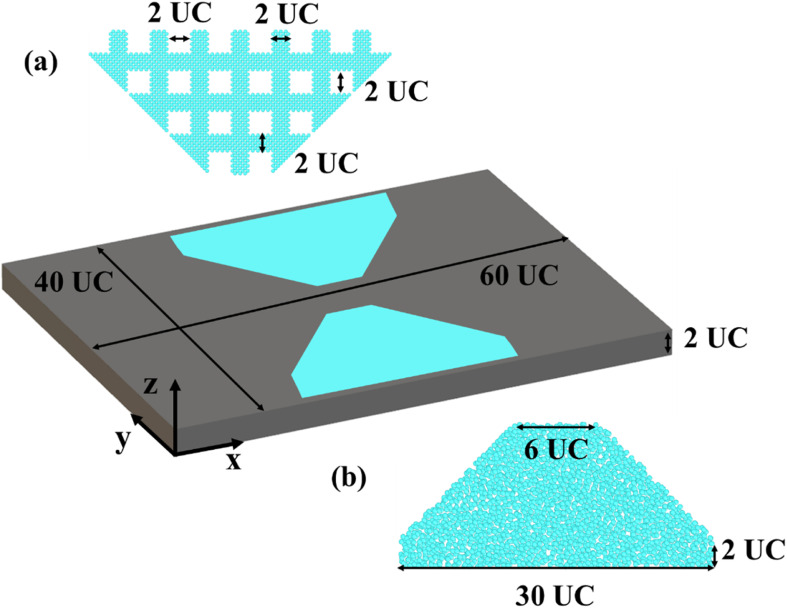
The atomistic models of the different heat flux concentrators. (a) The functional region of a perforated concentrator. (b) The functional region of an amorphous concentrator.

We show the atomistic models of the heat flux concentrator in Fig. 1. To achieve perfect surfaces, we cleave bulk Si supercells in the direction of [001], with the lattice constant of Si being 5.431 Å. A Si cubic unit cell (UC) is duplicated to create the nanofilm. The pristine nanofilm is made up of 60, 40, and 2 UC in the *x*, *y*, and *z* directions, respectively. As shown in Fig. 1, the grey section is the perfect silicon nanofilm, while the light blue parts are the functional sections. In this work, we consider two ways to construct the functional sections: the perforated concentrator (using patterned nanoholes); the amorphous concentrator (using amorphized silicon). For the perforated concentrator, the side width of the nanohole is 2 × 2 UC, with a spacing of 2 UC. For the amorphous concentrator, to generate the amorphous region, we start with the crystalline Si. We use the Nosé–Hoover thermostat^46^ to heat the crystalline Si from 300 K to 4000 K and equilibrate it at 4000 K (above the melting point) for 100 ps to achieve the amorphous state. Then it is quickly annealed to 300 K with a cooling rate of 185 K ps^−1^. This approach has been used to generate amorphous Si in previous studies.^47–50^ We refer to both nanoholes and amorphous regions as functional regions. For comparison, the pristine film is also considered.

We use the LAMMPS packages^51^ to conduct the non-equilibrium MD simulation to investigate the heat flux concentration of the different concentrators. The Stillinger–Weber potentials^52^ are used to describe the interactions between Si atoms in all MD simulations. At the start of the simulations, the system is first energy minimized, and then all atoms are set at 300 K with Gaussian distribution velocities. In the canonical ensemble (which keeps the number of atoms, volume, and temperature constant, also called NVT), the system is relaxed for 100 ps. Next, we fix the atoms in the region of 2 UC along the *x*-axis of both ends and use the Nosé–Hoover thermostat to establish a temperature gradient along the *x*-axis by placing the atoms in the thermostat region (including a hot bath and a cold bath, and their lengths are both 8 UC) at 320 K and 280 K, respectively. Finally, except for the thermostat and fixed regions, the system is placed in the micro-canonical ensemble (which keeps the number of atoms, volume, and energy constant, also called NVE) for MD simulations. It is conducted for 5 ns, after which the system reaches a nonequilibrium steady state within 2 ns.

## 3 Results and discussion

### 3.1 Heat current concentrator with a patterned amorphous section

**Fig. 2:**
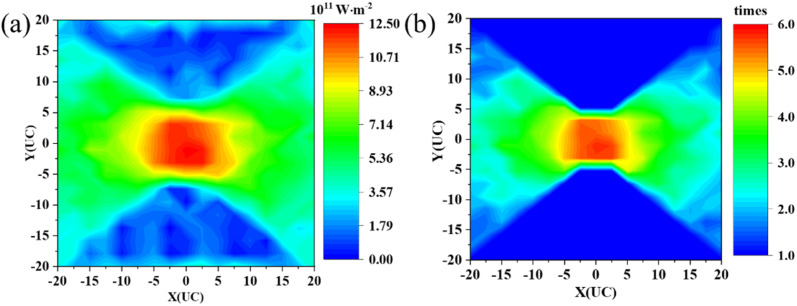
(a) The heat flux profile of the patterned amorphous concentrator. (b) The ratio of heat flux (RHF) of the patterned amorphous concentrator.

During the simulations, we divide the system into 25 × 20 small blocks and calculate their heat flux and temperature. The heat flux profile of the amorphous concentrator is illustrated in Fig. 2(a) and other results are shown in Fig. S1 and S2.† The pristine film has a uniform temperature distribution from the hot to the cold bath, and the heat flux is distributed relatively uniformly along the *x*-axis, demonstrating that the system is in a nonequilibrium steady state. However, in the amorphous concentrator, the presence of a patterned amorphous section leads to a very different temperature distribution, with an increase in temperature along the *x*-direction near the amorphous region, creating a temperature gradient and driving the heat flux towards the central region. As a result, the local heat flux in the center is extremely high. Therefore, a heat flux concentrator is realized using this patterned nanophononic metastructure. In addition, we calculate the temperature gradient along the *x*-direction, as shown in Fig. S3.† The temperature gradient is uniformly distributed for the pristine film. However, the presence of the amorphous structure leads to a larger temperature gradient in the central region.

To quantitatively evaluate the ability of heat flux concentration, we define the ratio of heat flux (RHF) as RHF = *J*/*J*_functional_, where *J* is the local heat flux and *J*_functional_ is the average heat flux within the functional region as a reference. The RHF distribution of the amorphous concentrator is shown in Fig. 2(b). The RHF distribution profile is the same as the heat flux distribution, with the largest RHF in the central region, and the heat flux in the central region can reach 6 times that in the adjacent region.

Previously, temperature concentration was obtained by setting up core–shell^53–55^ or multilayer structures^56,57^ through transformation thermotics theory, effective medium theory,^58^ and topological optimization methods.^59^ However, the heat flux concentration ratio is not high. For example, Yu *et al.*^53^ designed a heat flux concentrator, in which the heat flux in the central region is about 2.6 times that of the background region. In the amorphous concentrator studied in the present work, remarkable heat flux concentration is realized due to the reduced thermal conductivity in the amorphous region.^60–62^

### 3.2 Heat flux concentrator based on a nanomesh structure

**Fig. 3:**
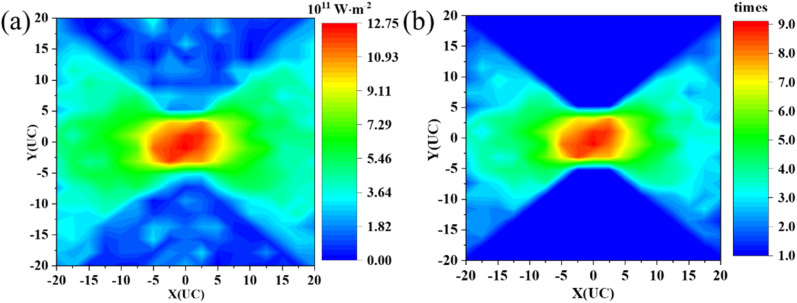
(a) The heat flux profile of the perforated concentrator. (b) The ratio of heat flux (RHF) of the perforated nanomesh concentrator.

In recent years, nanomesh structures have shown promising potential for thermoelectric applications due to their low thermal conductivity and good electrical properties. In this section, we investigate the application of nanomesh structures to heat flux concentration. Again, we start by analyzing the heat flux and temperature profile of the perforated concentrator. As shown in Fig. 3(a), the perforated concentrator has a similar heat flux profile to the patterned amorphous sample. The local heat flux in the center is also extremely high. The main reason for this is the presence of nanomesh, which severely impedes the transfer of heat flux. This indicates that heat flux concentration can be achieved by arranging the nanomesh structure. Furthermore, we calculate the RHF of the nanomesh concentrator. Similarly, the average heat flux within the nanomesh region is set as a reference. We present the RHF distribution of the nanomesh concentrator in Fig. 3(b). The heat flux in the central region can be up to 9 times that in the adjacent region. Clearly, this is much higher than that in the amorphous concentrator, indicating that the arrangement of nanomesh is more effective in achieving heat flux concentration. The temperature gradient along the *x*-direction is shown in Fig. S3,† where the perforated nanomesh has an obvious non-uniform temperature gradient distribution.

This is mainly because the nanomesh structure introduces a quadratic artificial periodicity, which may change the phonon dispersion relation and result in a reduction in the phonon bandgap, phonon density of states, and group velocity, which can suppress the thermal conductivity significantly.^63,64^

### 3.3 Phonon localization and heat flux regulation

In the aforementioned sections, we found that patterned amorphous and nanomesh structures can produce heat flux concentration and that nanomesh structures have higher heat flux concentration ability. In amorphous materials, a large number of heat carriers are spatially localized modes; thus, their contribution to heat conduction is significantly suppressed. Even delocalized diffusion, as they contribute to heat conduction in a diffusive manner, also results in low thermal conductivity.^62^ In a nanomesh structure, the artificial periodicity can modify the phonon dispersion relation, induce a decrease in phonon group velocity and thus suppress the local thermal conduction.^63^ Overall, the functional regions constructed by patterned amorphous or nanomesh structures reduce the local heat flux and eventually lead to a concentration of heat flux in the central region. Next, we use phonon localization theory to further explore the underlying physical insights.

In the different structures, we choose the same region (except for the thermostat and fixed region) for calculating the velocity autocorrelation function, and the autocorrelation time is 30 ps. The phonon density of states (PDOS) can be calculated using the Fourier transform of the velocity autocorrelation,^65^1
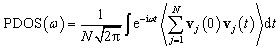
where *ω* is the phonon frequency, *N* is the number of atoms, and **v** is the velocity vector.

**Fig. 4:**
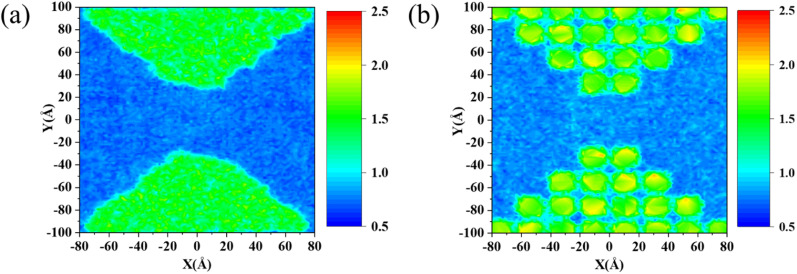
The intensity of localized phonon mode in different concentrators, (a) the amorphous concentrator and (b) the perforated concentrator.

The mode participation rate (MPR) can provide detailed information on the effect of localization; it can be calculated based on the PDOS,^66^2
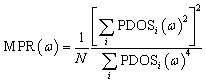
where PDOS_*i*_ (*ω*) is the phonon density of states at specific atomic *i* locations according to eqn (1). However, our interest is focused on regions that generate localization. Therefore, to obtain specific position information about phonon localization, we further calculate the intensity of localized modes (*Λ* ∈ MPR < 0.4),^67,68^ as follows:3
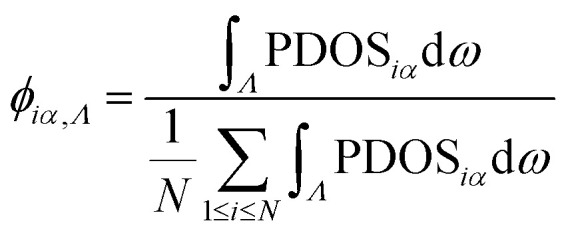
where *α* = *x*, *y*, and *z*. In the calculation, we average *ϕ*_*iα*_ over all atoms with the same *x*/*y* and plot the intensity of the localized phonon modes in the *xy*-plane. The higher the value of *ϕ*_*iα*_, the stronger the localization of the phonon mode at the *i*th atom. The MPRs of the pristine film and amorphous and perforated nanomesh concentrators are shown in Fig. S4,† where the MPRs of both amorphous and perforated nanomesh concentrators are reduced compared to that of the pristine film. The intensity of localized phonon modes of amorphous and perforated concentrators is shown in Fig. 4(a) and (b), respectively. Clearly, the localized modes are distributed in the functional region. These results provide a direct demonstration that phonon localization takes place within the functional regions. The amorphous regions all produce extremely strong localization, while the nanohole regions produce extremely strong localization for each hole, and the hole gaps also produce localization due to the proximity effect, but the localization is weaker.

**Fig. 5:**
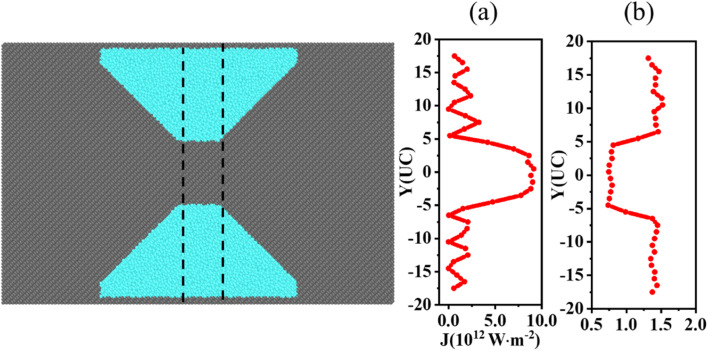
(a) The heat flux spatial distribution of the amorphous concentrator. (b) The distribution of intensity of localized phonon modes of the amorphous concentrator.

Finally, we select the same region along the *x*-axis in both concentrators and analyze the spatial distribution of heat fluxes and localized phonon modes within the region. The location-dependent heat flux and localized phonon mode distribution of the amorphous concentrator are shown in Fig. 5(a) and (b), respectively. The heat flux distribution in the same region of the pristine film is shown in Fig. S5.† For pristine films, the heat flux is uniformly distributed along the *y*-axis. As in Fig. 5(a), the central region of heat flux along the *y*-axis of the amorphous concentrator is much higher than the edge region. And, as shown in Fig. 5(b), the localized phonon modes along the *y*-axis are much lower in the central region than in the edge region. The more localized modes, the stronger the obstruction to heat flux, indicating that the localization of phonon modes in the functional region is the main reason for the heat flux concentration.

**Fig. 6:**
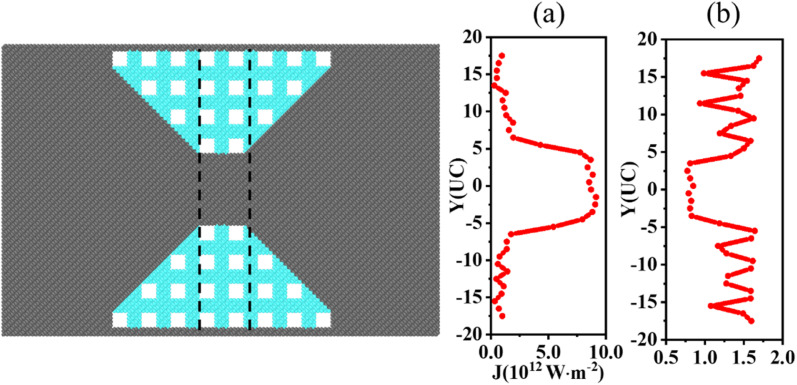
(a) The heat flux spatial distribution of the perforated concentrator. (b) The localized phonon modes spatial distribution of the perforated concentrator.

In Fig. 6(a) and (b), we show the location-dependent heat flux and intensity of localized phonon modes of the perforated concentrator, respectively. The spatial distributions of the perforated and amorphous concentrators' heat fluxes are similar, with the central region being much higher than the edges. However, the strong Bragg scattering produced by the nanoholes results in non-uniform heat flux in the edge regions. At the same time, the spatial distribution of the localized modes also shows a non-uniform distribution at the edges and is smaller for larger heat fluxes. The Bragg scattering and strong phonon localization produced by the nanoholes ultimately lead to the heat flux concentration.

## 4 Conclusion

In summary, through molecular dynamics calculations, we design two heat flux concentrators using amorphous- and nanomesh-based nanophononic metastructures, respectively. We found that the heat fluxes in the central region of both concentrators are much higher than that in the adjacent regions. Furthermore, a more efficient concentration can be generated by the patterned nanoholes due to the stronger phonon localization. The underlying mechanisms are discussed based on phonon localization theory and the spatial distribution of localized modes. This work can reveal important applications in nanoscale heat flux regulation.

## Data availability

The data that support the findings of this study are available from the corresponding author upon reasonable request.

## Conflicts of interest

There are no conflicts to declare.

## Supplementary Material

NA-005-D3NA00494E-s001
