# Supplementary material for: Heat flux concentrators based on nanoscale phononic metastructures
Source: Nanoscale Adv. 2023 Sep 11;5(20):5641–8. doi: 10.1039/d3na00494e (PMC10563830; doi:10.1039/d3na00494e)
Supplement: NA-005-D3NA00494E-s001 [file NA-005-D3NA00494E-s001.pdf]

Electronic Supplementary Material (ESI) for Nanoscale Advances.
This journal is © The Royal Society of Chemistry 2023

Supplemental Information

Heat flux concentrator based on nanoscale phononic metastructures

Jian Zhang [1,2], Haochun Zhang [1,*], Weifeng Li[3], Gang Zhang [2,*]

[1] School of Energy Science and Engineering, Harbin Institute of Technology, Harbin 150001, China

[2] Institute of High Performance Computing, Agency for Science, Technology and Research (A*STAR), Singapore 138632, Singapore

[3] School of Physics & State Key Laboratory of Crystal Materials, Shandong University, Jinan 250100, Shandong, China

Figure S1 illustrates the temperature and heat flux profile of the pristine film. Figure S2 shows the temperature profiles of (a) the amorphous and (b) perforated concentrators.

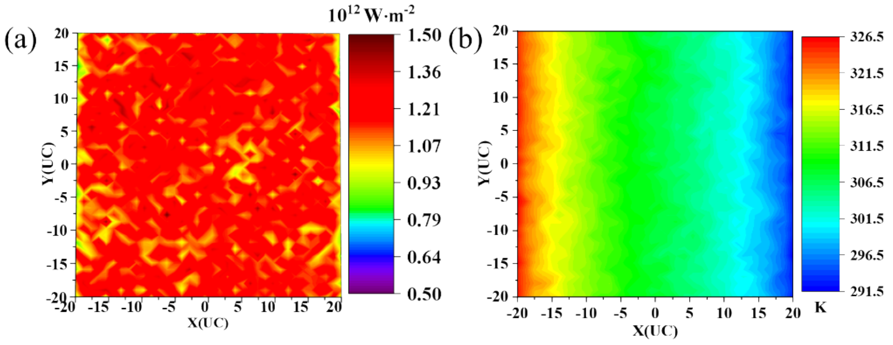

Figure S1. The heat flux (a) and temperature (b) profiles of the pristine film.

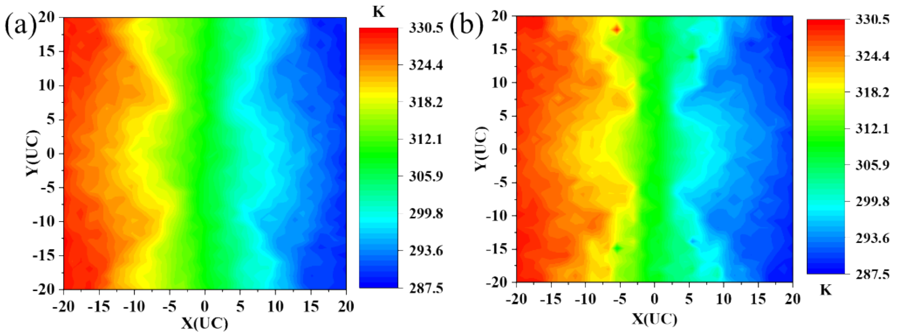

Figure S2. The temperature profiles. (a) the amorphous concentrator, (b) the perforated concentrator.

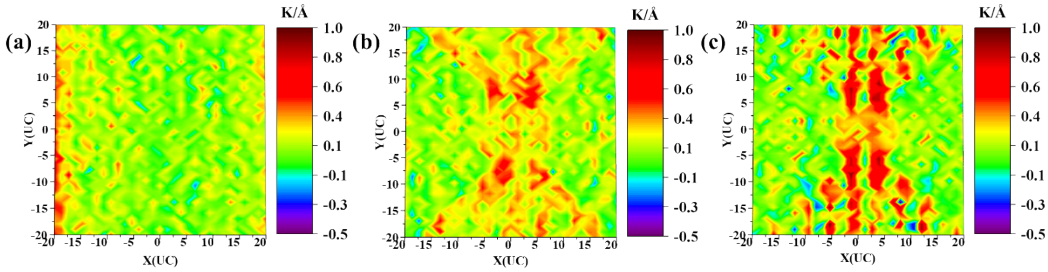

Figure S3. The temperature gradient along the x-direction. (a) the pristine film, (b) the amorphous concentrator, (c) the perforated nanomesh concentrator.

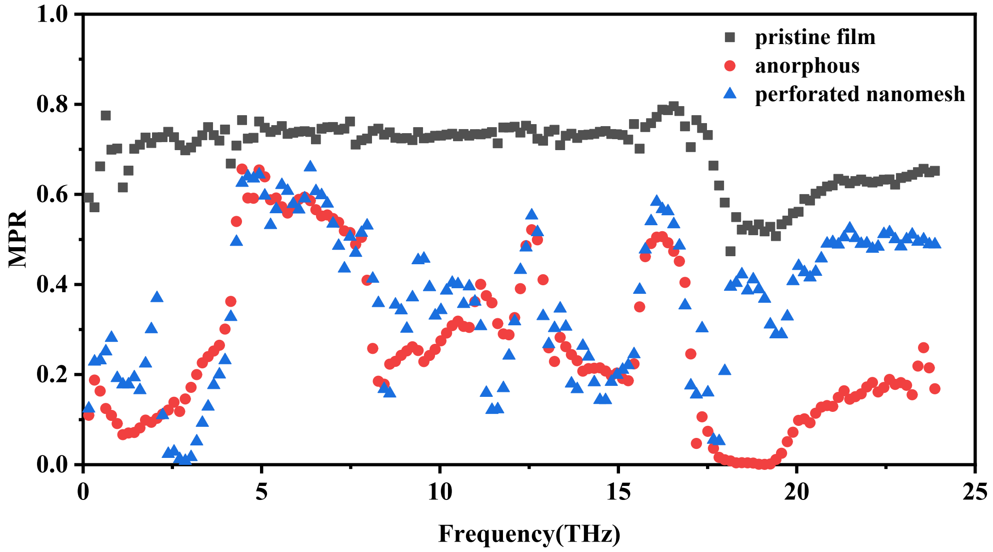

Figure S4. The MPRs of the pristine film, perforated nanomesh, and amorphous concentrators.

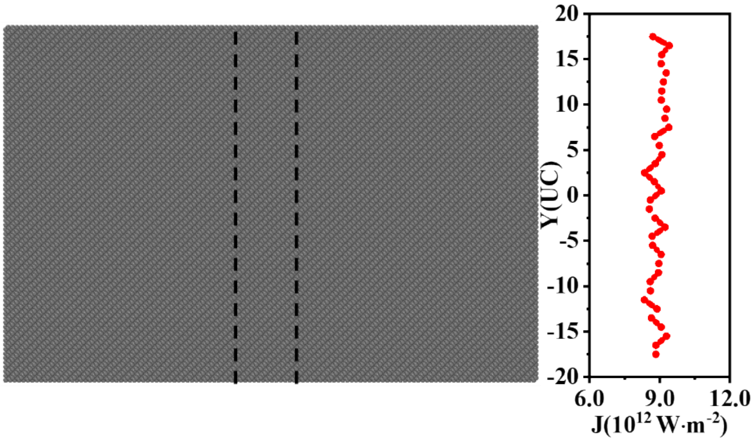

Figure S5. The heat flux spatial distribution of the pristine film.
